# A resilience-oriented approach for quantitatively assessing recurrent spatial-temporal congestion on urban roads

**DOI:** 10.1371/journal.pone.0190616

**Published:** 2018-01-02

**Authors:** Junqing Tang, Hans Rudolf Heinimann

**Affiliations:** ETH Zurich, Future Resilient Systems, Singapore-ETH Centre, Singapore, Singapore; Beihang University, CHINA

## Abstract

Traffic congestion brings not only delay and inconvenience, but other associated national concerns, such as greenhouse gases, air pollutants, road safety issues and risks. Identification, measurement, tracking, and control of urban recurrent congestion are vital for building a livable and smart community. A considerable amount of works has made contributions to tackle the problem. Several methods, such as time-based approaches and level of service, can be effective for characterizing congestion on urban streets. However, studies with systemic perspectives have been minor in congestion quantification. Resilience, on the other hand, is an emerging concept that focuses on comprehensive systemic performance and characterizes the ability of a system to cope with disturbance and to recover its functionality. In this paper, we symbolized recurrent congestion as internal disturbance and proposed a modified metric inspired by the well-applied “R4” resilience-triangle framework. We constructed the metric with generic dimensions from both resilience engineering and transport science to quantify recurrent congestion based on spatial-temporal traffic patterns and made the comparison with other two approaches in freeway and signal-controlled arterial cases. Results showed that the metric can effectively capture congestion patterns in the study area and provides a quantitative benchmark for comparison. Also, it suggested not only a good comparative performance in measuring strength of proposed metric, but also its capability of considering the discharging process in congestion. The sensitivity tests showed that proposed metric possesses robustness against parameter perturbation in Robustness Range (RR), but the number of identified congestion patterns can be influenced by the existence of *ϵ*. In addition, the Elasticity Threshold (ET) and the spatial dimension of cell-based platform differ the congestion results significantly on both the detected number and intensity. By tackling this conventional problem with emerging concept, our metric provides a systemic alternative approach and enriches the toolbox for congestion assessment. Future work will be conducted on a larger scale with multiplex scenarios in various traffic conditions.

## Introduction

Continuous growth of motor vehicles has made urban congestion become a serious national problem, one which has been receiving considerable attention from engineers, planners, researchers, and policymakers. The congestion issue in urban areas has always intertwined with other concerns which significantly affect our quality of life, such as air quality, urban noise, energy use, road safety and economic growth [1]. Traditionally, congestion can be categorized as recurrent and non-recurrent (incident-based). Apart from the latter, recurrent congestion influence road operation in a significant way and contribute to a large portion of urban traffic delay [2, 3] and its quantitative characterization has always been important for managing traffic in the urban context.

Studies about quantifying congestion abound, by and large, in literature as people attempted difference approaches to address it. For instance, measures with statistical perspectives were investigated by Federal Highway Administration. Lindley [4] promoted and studied the effectiveness of potential solutions to congestion. The statistical analysis indicates that the demand reduction strategies should be effective when looking for potential solutions. The study provides a first cut at estimating cost and congestion reduction potential giving available options. Moreover, Highway Performance Monitoring System (HPMS) [5] provided a solid database for statistical analysis of congestion. The work also estimates an aggregated impact of several techniques for reducing freeway congestion. D’abadie and Ehrlich [6] discussed various approaches for quantifying congestion and their effectiveness. They also compared two measures of congestion (distance-based and time-based) to describe the magnitude of congestion in a case study of New Jersey counties. The results showed that the time-based approach is more likely to have a high impact as it effectively provides a different perception of congestion and also a stronger guidance on major issue identification. Also, Milojevic and Rakocevic [7] proposed an algorithm, VANET, to enable vehicles in the network to be aware of the level of traffic congestion in a distributed way. The work tackles the congestion issue by enhancing the vehicle information communication to prevent early-form congestion and provides overall knowledge about congestion to drivers. On the other hand, Armah et al [8] attempted to study congestion and one of its side effects, air pollution, with a systemic approach. They provided overall systemic thinking flowcharts on urban congestion issue. But the assessment was largely qualitative. Kerner et al [9–11] conducted a series of deep investigation on bottleneck congestion and proposed a three-phase traffic theory for controlling and tracking spatial-temporal congestion in highway traffic patterns. There are many others and readers can refer to an incomplete list includes: [12–15]. With such ample options of various methods and approaches, an investigation emphasized on comprehensive systemic perspective is still missing for quantitative congestion assessment.

The concept of resilience originated in engineering mechanics, which can be retrospected back to the early 19th Century [16] and currently found in a wide range of areas [17] including engineering systems [18], ecology [19], psychology [20, 21], social science [22] and so forth. Even though the concept is still struggling for reaching an agreed definition [23], it is most commonly described as the ability of a system to cope with disturbance and recover its functionality afterwards [24]. In this way, Bruneau et al [25–27] proposed a quantitative framework to assess system resilience with “Resilience-Triangle” based on the level of functionality performance, and this is the so-called “R4” framework (Robustness, Redundancy, Resourcefulness, and Rapidity). They argued that resilience loss of system functionality can be assessed by calculating the area of the triangle on time-series performance. A large area of triangle denotes a less resilient system functionality. For congestion occurred in traffic flow it has the similar preference. However, some of its fundamental dimensions should be adjusted for traffic congestion studies.

A key differentiation with previous proposals is that the quantification of congestion was addressed, in this paper, using a new-build resilience-based metric that consists multiple dimensions and combined with emerging concept to provide a novel solution for the conventional issue. Our criterion bases on rethinking an urban highway as an integrated system and traffic quantities as its functionality indications. Hence, we examined and improved the “R4” framework, and adopted the “triangle” idea to quantify the congestion with a resilience-oriented approach in spatial-temporal performance.

## Materials and methods

### Data descriptions and conceptual discrete platform

**Fig 1:**
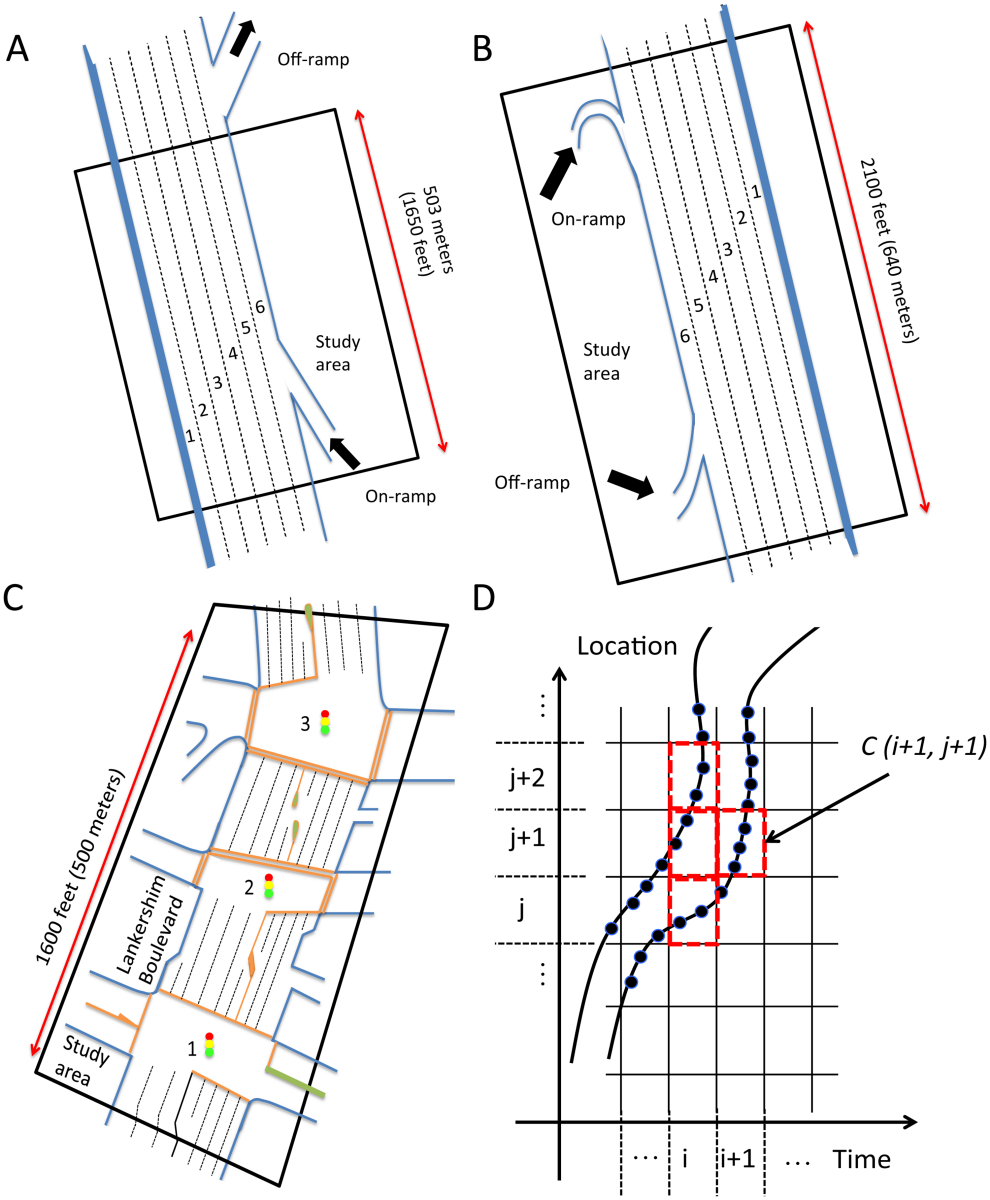
The study areas (not in scale) and discrete platform. Aerial view of lane configuration of (A) I-80; (B) US-101; and (C) Lankershim Boulevard, LB. (D) Conceptual cells constructed for spatial-temporal analysis.

All three datasets used for numerical studies were collected by the Next Generation Simulation Programme from the United States Federal Highway Administration [28]. The datasets contain detailed time-resolution vehicle trajectory information, including trajectory location, time, speed, acceleration, etc. Here, traffic in the first dataset was monitored on eastbound Interstate 80 (I-80) in the San Francisco Bay area near Emeryville, CA, on 13 April 2005. The study area is 1650 feet (approx. 503m) long and comprises six freeway lanes that include one heavy-goods vehicle (HGV) lane and one on-ramp (Fig 1A). The full dataset covers a span of 45 minutes in total and is segmented into three 15-minute subsets, i.e., 16:00 p.m. to 16:15 p.m., 17:00 p.m. to 17:15 p.m., and 17:15 p.m. to 17:30 p.m.

The vehicle trajectory data from the second dataset was collected on southbound of freeway US-101, also known as the Hollywood Freeway in Los Angeles, on 15 June 2005. The study area is approximately 2100 ft (approx. 640m) in length and consists of five mainline lanes throughout the section and one auxiliary lane as lane 6 (Fig 1B). A total of 45 minutes of data from morning peak is segmented as well into three 15-minute periods: 7:50 a.m. to 8:05 a.m.; 8:05 a.m. to 8:20 a.m.; and 8:20 a.m. to 8:35 a.m. The first two freeway cases all contain various vehicle types, and because normal traffic in the HGV lane and the ramps differ from that found in the other lanes, they were eliminated from our consideration. More details of these two study areas can be found in [29, 30].

Having first two cases selected from freeway vehicle data, the third dataset was from a section of an urban arterial. The data was collected on Lankershim Boulevard (LB) in the Universal City neighborhood of Los Angeles, CA, on 16 June 2005. This arterial area covers three signalized junctions and is about 1600 ft (approx. 500m) in length, which contains three to four lanes in dual-way directions (Fig 1C). The observation period was 30 minutes in total: 8:30 a.m. to 8:45 a.m. and 8:45 a.m. to 9:00 a.m during morning peak hours. The data contains various vehicle types and different lane layout and there is no special vehicle or lane types excluded for analysis of this case, whereas the main portion of traffic was still passenger vehicles [31]. Table 1 summarizes the basic information about all datasets. Because we would like to capture the steady and comprehensive patterns and also to avoid inactive cells in spatial-temporal profiles, the first and last 150 seconds in the temporal dimension and 100 feet (approx. 30.5m) in the spatial dimension were removed.

**Table 1:** Brief summary of datasets [29–31].

ID & Type	Time span	Direction	Length, vehicle, and lane types
I-80 Freeway	45 mins 16:00 p.m. to 16:15 p.m. (working hours) 17:00 p.m. to 17:30 p.m. (evening peak hours)	Eastbound	1650 ft Four passenger-vehicle lanes with one HGVs and one ramp lanes Freeway lanes WITHOUT signal control
US-101 Freeway	45 mins 7:50 a.m. to 8:35 a.m. (morning peak hours)	Southbound	2100 ft Five passenger-vehicle lanes and one ramp lane Freeway lanes WITHOUT signal control
Lankershim Boulevard (LB) Urban street	30 mins 8:30 a.m. to 9:00 a.m. (morning peak hours)	Dual way	1600 ft Three to four main lanes for mixed Passenger vehicles, Trucks, and Motorcycles Three to six lanes at junctions WITH signal control junctions

In Fig 1D, development of the conceptual platform begins with establishing the discrete cells. The study areas were depicted into cells with dimensions of 4 seconds × 70 feet (approx. 21.34m). We calibrated dimensions to ensure an efficient discretization. If, however, the cell is too small, then the number of vehicles in each cell would not be representative. Likewise, the propagation pattern of congestion would have been ambiguous if those cells were too large (see details in sensitivity test section). Because the spatial-related dimensions of raw data for this study were expressed using “foot” or “feet (ft.)” as the unit of measurement, our results applied the same unit to keep consistency. But where possible, those values have been converted into the International System of Units.

### Resilience-oriented approach

**Fig 2:**
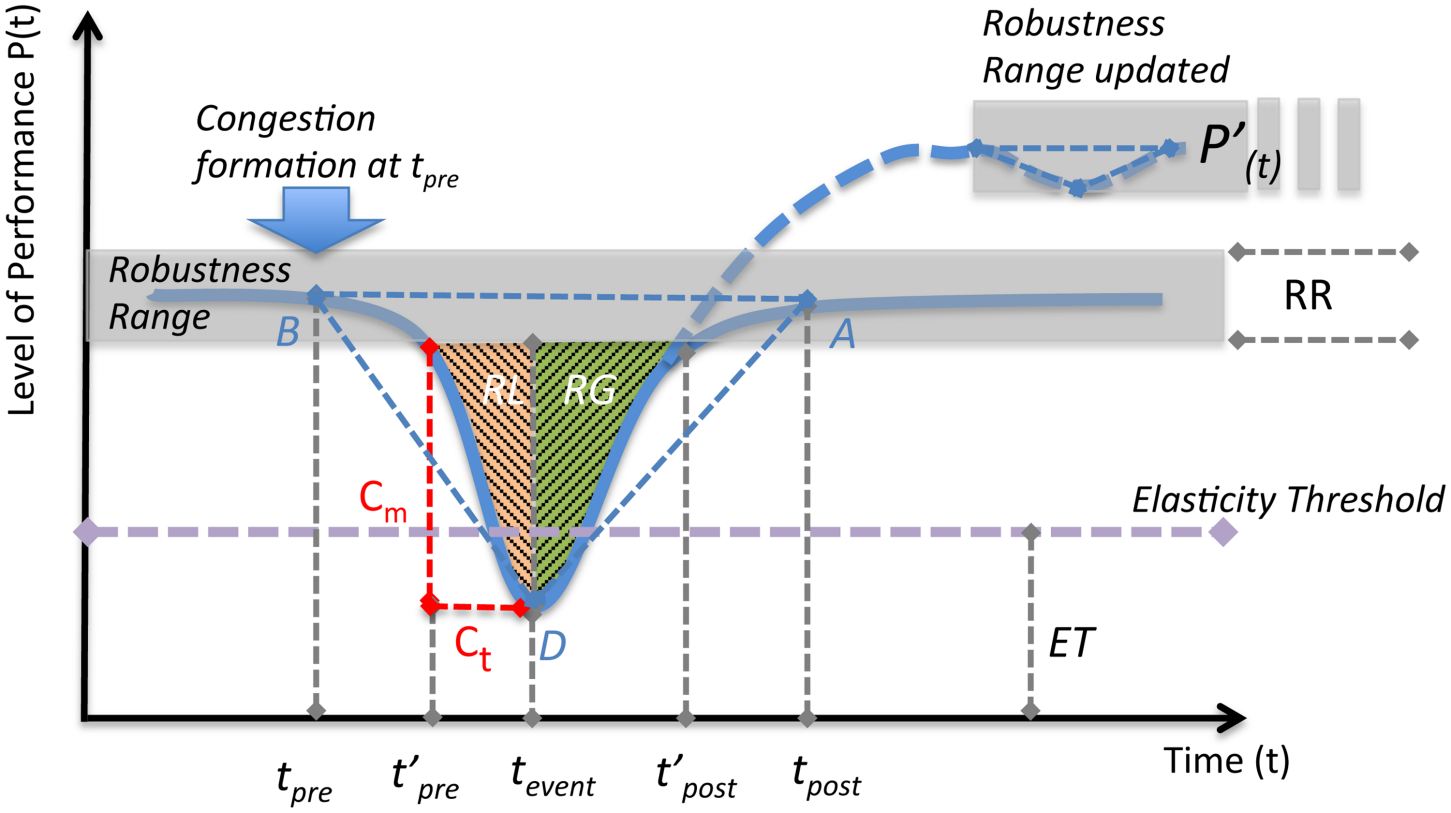
Typical draw-down and draw-up cycle. In this case, external shock occurs at time *t*_*pre*_ and the performance recovers at *t*_*post*_. Time-series performance is able to have several cycles as the process could be dynamic. The grey band is the Robustness Range, which can be dynamic and adaptive in each cycle as if in $P_{\left(t\right)}^{\prime }$. Δ*ABD* represents the “Resilience-triangle”. Colour-pattern shades denote the areas we consider in our quantification metric and fundamental dimensions are defined accordingly.

The performance of a system decreases after a shock, and if possible, recovers within a certain time. This can be observed in many cases, like formation and dissolution of congestion in traffic. Accordingly, the “R4” resilience-triangle metric [25] was proposed upon a very straightforward proxy: a system’s resilience loss is the loss of performance. And it is defined by (a) the draw-down line (the downturn section which starts from performance prior to shock to lowest level after the shock), (b) the draw-up line (the recovery section), and (c) the time period required for whole process (from the head of draw-down line to the end of draw-up line). A pair of down-and-up lines forms a draw-down and draw-up cycle. Thereby, it is convincing that using triangle’s area to represent the resilience loss is sensible (area of triangle Δ*ABD* in Fig 2). Nevertheless, we split this triangle into two segments, Resilience Loss (RL) and Resilience Gain (RG), since it would be more sensible to understand downturn and upturn separately. In this way, congestion can then be effectively represented by RL in time-series traffic performance.

Even the “R4” framework set a good paradigm to characterize system resilience, it overlooked the effects of different recovery paths and other essential fundamental dimensions. It is commonly held that there are four possible recovery paths that a system performance could behave: adaptive recovery, just recovery, insufficient recovery, and collapse. Hence we improve the framework and establish novel dimensions for congestion assessment as follows.

Given that:

- Function *P*_(*t*)_ represents performance behavior of just recovery, which is the normal case for a system performance. And $P_{\left(t\right)}^{\prime }$ is another possible recovery path with adaptive recovery.
- The head and the tail of draw-down section are expressed as *t*_*pre*_ (pre-event) and *t*_*event*_ respectively, and the successive draw-up terminates at time *t*_*post*_ (post-event).

We define the following fundamental dimensions in terms of congestion:

**Elasticity Threshold (ET)**: Similar to the concept of elasticity in material mechanics, traffic performance should have a threshold at which the self-organizing ability and free-flow state start to deteriorate. Variety of studies suggest the existence of phase transition in traffic status [32–34]. We assume that losing a mild amount of elasticity will result in performance being above the ET. Nevertheless, with an overdose of elasticity loss, the performance would fall below ET, then extra effort is needed to push it back into the region of elasticity. In this study, the values of ET were determined by critical density in traffic data (details on the determination of ET can be found in the following sections).

**Robustness Range (RR)**: Of particular note is the fact that a certain range of robustness ubiquitously exists (e.g., blood pressure is always monitored as acceptable within a certain range). General system performance naturally varies in time with tolerable fluctuations. Because the target is recurrent congestion, we need to identify the extent to which a decrement in performance can be considered as congestion rather than a random oscillation of the traffic. In principle, we assume the drop or raise, as long as it is in RR, are not effective in our quantification consideration. The width of the range is defined as 1/10 of ET in the analysis. Unlike ET fixed for entire time series, one should note that the RR in different cycles could be dynamically updated.

**Congestion Magnitude (*C*_*m*_)**: This is a straightforward dimension that indicates the extent to which recurrent congestion occurs. Of note is that half of RR should be ruled out from the calculation of *C*_*m*_ since only the amount of drop outside the RR would be effective for quantification purpose. Thus the effective draw-down starts at $t_{pre}^{\prime }$.

$$\begin{array}{c} C_{m}=P_{\left(t_{pre}^{\prime }\right)}-P_{\left(t_{event}\right)} \end{array}$$ (1)

**Congestion Time (*C*_*t*_)**: defined as ratio of congestion formation time to total cycle time. Similarly, because of the effect of RR the values of *C*_*t*_ should be adjusted, from $t_{pre}^{\prime }$ to *t*_*event*_.

$$\begin{array}{c} C_{t}=\frac{t_{event}-t_{pre}^{\prime }}{t_{post}^{\prime }-t_{pre}^{\prime }} \end{array}$$ (2)

**Recovery Scenario (*R*_*s*_)**: or the recovery ability, is the dimension that illustrates the recovery path in each draw-down and draw-up cycle. In order to differentiate major congestion (insufficient recovery or collapse, i.e., the congestion is discharged partially or never discharged) and other congestion (just and adaptive recovery, i.e., congestion is mitigated and discharged completely), we define the sign of *R*_*s*_: Negative (-) for insufficient recovery or collapse, and positive (+) for just and adaptive recovery. A large positive *R*_*s*_ means *P*_(*t*_*post*_)_ > *P*_(*t*_*pre*_)_, which denotes a severe congestion occurred but with a sufficient discharging process after its formation.

$$\begin{array}{c} R_{s}=\{\begin{array}{cc} & 1\;\;\text{if}\;P_{\left(t_{post}\right)}⩾P_{\left(t_{pre}\right)}\\ & -1\;\text{if}\;P_{\left(t_{post}\right)}<P_{\left(t_{pre}\right)} \end{array} \end{array}$$ (3)

**Resistance coefficient (*R*_*e*_)**: It is a quantity that characterizes the input effort for resisting the downturn tendency and is strongly associated with ET, i.e., if the minimum level drops below ET, *R*_*e*_ has a value greater than zero because a large amount of effort would be input to resist the drop and more effort would be needed to restore performance, and let *R*_*e*_ equals to zero when the minimum level is above the ET, that is, no phase transition occurs. Thus determination of *R*_*e*_ is positively related to the minimum level of performance *P*_(*t*_*event*)__. One may note that *R*_*e*_ has no interaction with the draw-up section as it is mainly described as a dimension from the draw-down section. This is because the effective resistance naturally happens during downturn process, lasting until performance reaches the minimum level, then it would be ready to recover after it.

$$\begin{array}{c} R_{e}=\{\begin{array}{cc} & ET-P_{\left(t_{event}\right)}\;\;\text{if}\;\;P_{\left(t_{event}\right)}<ET\\ & 0\;\text{if}\;\;P_{\left(t_{event}\right)}>ET \end{array} \end{array}$$ (4)

As mentioned, Δ*ABD* is split into RL and RG (Fig 2). General speaking, RL represents the cumulative effect of resilience loss in drawdown process (in our case, drawdown process denotes the formation process of congestion, because congestion is a type of performance loss in terms of traffic condition). Thus, by approximating the shaded areas as triangles the Congestion Index (CI) of a time-dependent observation can be expressed as:

$$\begin{array}{c} Congestion\;Index=\left(\frac{C_{m}\times C_{t}}{2}+R_{e}\right)\times R_{s} \end{array}$$ (5)

The rationale of Eq 5 is as follows: A recurrent congestion pattern can be depicted with two portions. One is the cumulative loss in its formation process, which is denoted as (*C*_*m*_ × *C*_*t*_)/2, and another is the jamming severity contributed by phase transition, which is *R*_*e*_. What’s next, these two portions are all associated with dynamic and repeating form-and-resolve process (draw-down and draw-up cycles). Thus the term *R*_*s*_ is brought into play to depict various recovery behavior in discharging process.

### Approaches based on travel time and volume-to-capacity ratio

Even there is no common definition of traffic congestion [35], many approaches and measures have been developed to scale its magnitude and intensity. Traditionally, two approaches are particularly popular and well-applied: travel time based and volume-to-capacity (V/C ratio) based. Two measures for metric comparison purpose are selected: Relative Congestion Index (RCI) and Level of Service (LoS).

RCI is conventionally defined as the ratio of delay time (DT) and free-flow travel time (*T*_*ff*_), which can be defined as [36]:

$$\begin{array}{c} RCI=\frac{DT}{T_{ff}}=\frac{T_{ac}-T_{ff}}{T_{ff}} \end{array}$$ (6)

where *T*_*ac*_ is the actual travel time needed. The RCI of zero denotes a very low level of congestion while values greater than two show significant congested states. Because our analysis is based on spatial-mean performance of the traffic, the *T*_*ac*_ and *T*_*ff*_ can be obtained also with spatial-mean quantities as:

$$\begin{array}{c} T_{ac}=\frac{Spatial\;length}{Spatial-mean\;speed} \end{array}$$ (7)

and

$$\begin{array}{c} T_{ff}=\frac{Spatial\;length}{Free-flow\;speed\;(v_{ff})} \end{array}$$ (8)

LoS approach is a more interpretable and straightforward measure to represent various static traffic states. As adopted in Highway Capacity Manual (HCM) [37], this method has become extremely popular in practice, especially for non-technical users [38]. The LoS can be determined by various traffic quantities, such as density, speed, V/C and maximum service flow rate. Rather than assigning quantitative values, the LoS assesses traffic conditions based on scale intervals (Table 2). The V/C ratio can be calculated as:

$$\begin{array}{c} V/C=\frac{Spatial-mean\;volume}{N_{max}} \end{array}$$ (9)

where, *N*_*max*_ is the maximum number of vehicles that one cell is able to contain, which represents the capacity. This term can be approximated by assuming an average vehicle length occupancy. We write:

$$\begin{array}{c} N_{max}=\frac{L_{cell}}{L_{occupancy}}\times N_{lanes} \end{array}$$ (10)

**Table 2:** Level of Service (LoS) and its corresponding V/C ratio and traffic states [37].

LoS class	Traffic state and condition	V/C ratio
A	Free flow	0∼0.60
B	Stable flow with unaffected speed	0.61∼0.70
C	Stable flow but speed is affected	0.71∼0.80
D	High-density but stable flow	0.81∼0.90
E	Traffic volume near or at capacity level with low speed	0.91∼1.00
F	Breakdown flow	>1.00

*L*_*cell*_ is the spatial length of cells, *N*_*lanes*_ is the number of lanes and *L*_*occupancy*_ is the average vehicle length occupancy and it comprises two parts: vehicle length *L*_*v*_ and safety distance *L*_*s*_. Because it is normally assumed that *L*_*v*_ is about 14 ft. (approx. 4.27m) [39], we assume *L*_*occupancy*_ is about 15 ft. (approx. 4.57m). The *N*_*lanes*_ is four in I-80 and five in US-101, recall that the HGV and ramp lanes are not considered, and we take 4.5 for the number of lanes in both northbound and southbound direction on LB to average its various lane layout through sections and at junctions. Once the V/C ratio is obtained, the LoS can be determined according to Table 2.

Although both measures are widely adopted in various studies, they unavoidably possess some weaknesses and disadvantages [38]: Firstly, for RCI approach it has been argued that the ratio is limited and heavily relied on particular road type and facility. Secondly, for LoS approach, it cannot provide a continuous range of values to represent the intensity of congestion.

## Results

In this section, the proposed metric is implemented and tested in empirical studies. Comparisons of measuring strength and metric sensitivity are investigated as well. Having all data descriptions, testbed setup and methodological frameworks constructed and outlined, next following step act as a guideline for readers to well understand the entire experimental procedure and to facilitate further analysis.

**Step 1.** **Understanding the traffic data:** An unambiguous and fundamental properties of data must be obtained, such as critical density, jam density, and free-flow speed.

**Step 2.** **Selecting appropriate Key Performance Indicator (KPI) and preparing the spatial-temporal profiles:** The resilience-oriented approach is performance-based and an appropriate KPI is needed for indicating various performance levels. Also, the spatial-temporal traffic patterns are obtained for exploratory analysis.

**Step 3.** **Denoising, normalizing and identifying filtered draw-down and draw-up cycles:** In this step, we need to de-noise and normalize the selected KPI first and then identify reasonable forming-and-discharging congestion cycles.

**Step 4.** **Estimating values for Elasticity Threshold (ET) and Robustness Range (RR):** These parameters need to be set next since many elemental functions in proposed metric rely on these two parameters.

**Step 5.** **Implementing metrics and further analysis:** Calculation and measuring results are conducted and further sensitivity tests analyses are presented.

### Jam density, critical density and free-flow speed

With discrete cells conceptualized on the study area, the first-order traffic quantities, density, speed and flow, can then be determined. The density *k*_(*i*,*j*)_ within each cell *C*_(*i*,*j*)_ was computed as *k*_(*i*,*j*)_ = *n*_(*i*,*j*)_/*l*_(*i*,*j*)_, where *n*_(*i*,*j*)_ denotes the number of vehicles in cell *C*_(*i*,*j*)_ at time *i* at *j* location, and the *l*_(*i*,*j*)_ is the spatial length of the cell, which in this case is a fixed term of 70 ft (approx. 21.34m).

**Fig 3:**
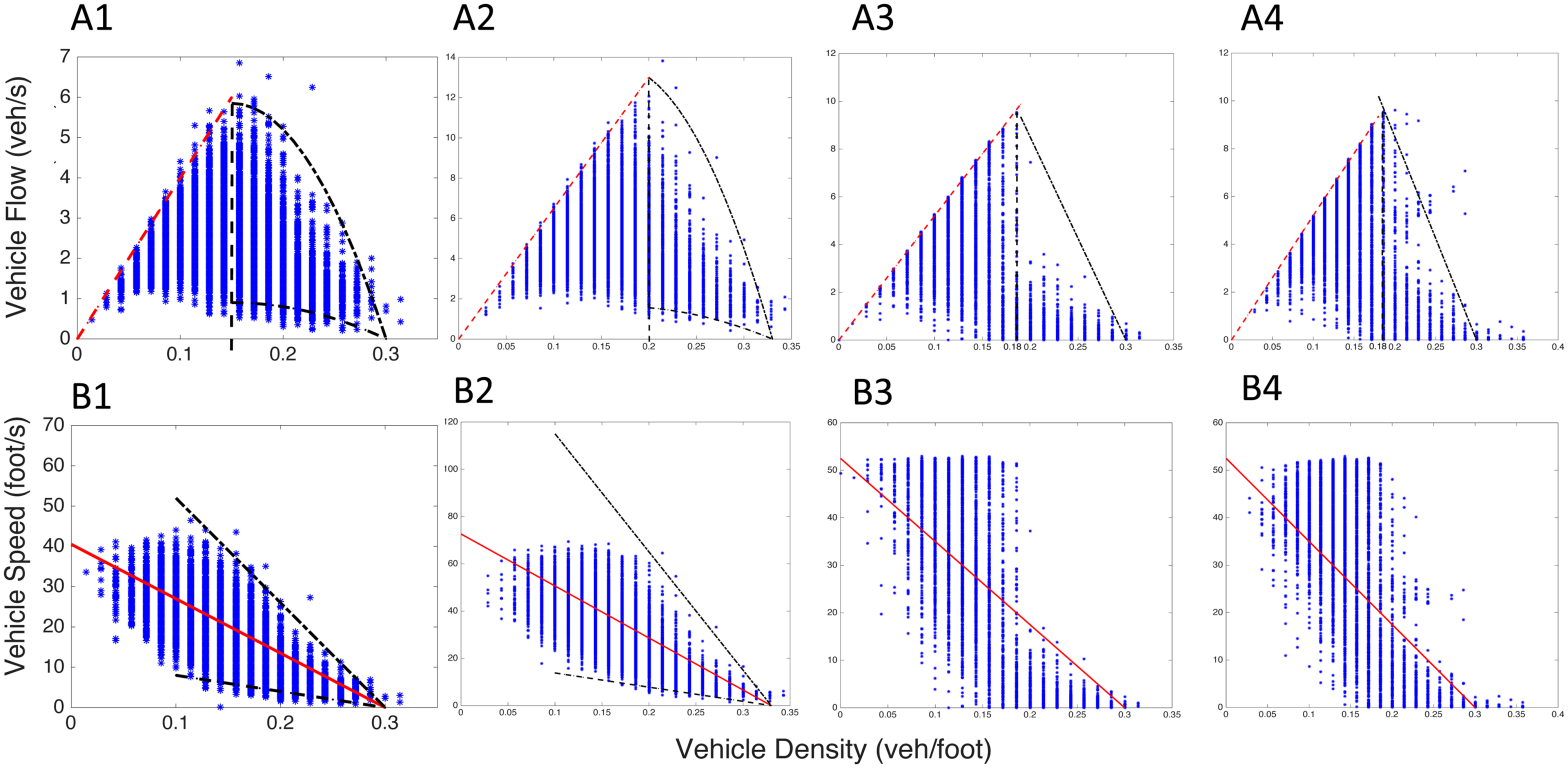
Density-flow and density-speed relationship of fundamental diagrams. (A) Density-flow relationship. (B) Density-speed relationship. (1) Case I-80. (2) Case US-101. (3) Northbound of LB, and (4) Southbound of LB. Red line: linear regression model; Red dotted line: linear approximation in free-flow phase with a slope as free-flow speed, which can also be determined by maximum speed in density-speed plots. Black dotted line: envelopes constructed for data containment of more than 95%, and the vertical black dotted line is the location of estimated critical density.

The dataset also contains speed information at each trajectory point. Thus the *v*_(*i*,*j*)_ was estimated by taking the average speed of all trajectory points in *C*_(*i*,*j*)_. The flow in that cell was calculated as the product of the speed and the density *q*_(*i*,*j*)_ = *k*_(*i*,*j*)_ × *v*_(*i*,*j*)_. In Fig 3A1, *k*_*jam*_ is roughly estimated as 0.30 veh/ft for I-80. To verify this, we applied a linear regression model to its density-speed plot (Fig 3B1) and found that the intersection with x-axis accredits the estimation of jam density. In this way, the jam density for US-101 case can be approximated as 0.33 veh/ft, and 0.30 veh/ft for both northbound and southbound in LB case.

The critical density, *k*_*critical*_, can be determined in each density-flow relationship plots as well. It lies on the point when traffic state transforms from free-flow phase to congestion phase (Let us only consider traditional two-phase traffic theory here for simplification. The three-phase traffic theory [40] will not be discussed). Therefore, it was estimated by finding the crossing point of linear approximation in free-flow phase and upper envelope in congestion phase while keeping a high data containment. In addition, the slope of this linear approximation in free-flow phase is the free-flow speed *v*_*ff*_, or forward wave speed. This quantity can be verified by the maximum speed fitted in linear regression in the density-speed relationship. Thus, the *k*_*critical*_ and *v*_*ff*_ were estimated as 0.15 veh/ft and 40 ft/s for I-80, 0.20 veh/ft and 65 ft/s for US-101, 0.18 veh/ft and 52 ft/s for both northbound and southbound in LB, respectively.

### Key Performance Indicator (KPI) and spatial-temporal density performance

Next, we need to illustrate overall performance with appropriate measurement. Those measurements identify current performance states of the system and act as indications on how and where the gaps between current and desired Level of Performance (LoP) [41]. Key Performance Indicator (KPI) is a unique or a set of performance measurements which is deliberately selected for representing LoP [42]. The selection criteria should ensure that (1) selected KPI can be tied into the overall study purpose and goals; (2) the KPI should directly reflects the LoP changes over time; (3) the KPI should allow you to establish measurable tracks for management.

In our cases, we used aggregated spatial-mean density capacity as KPI. It denotes spatial-mean capacity of a road section to accommodate traffic, and can be defined as $k_{\left(i\right)}^{\prime }=k_{jam}-{\bar{k}}_{\left(i\right)}$, where ${\bar{k}}_{\left(i\right)}$ is the spatial-mean density of study area at time *i*. We selected this density capacity as KPI for recurrent congestion as it is a direct, measurable and representative indicator for traffic, i.e., drops of this KPI indicate system performance loss as decreasing density capacity represents formation of congestion, which has consistent logic with proposed metric.

**Fig 4:**
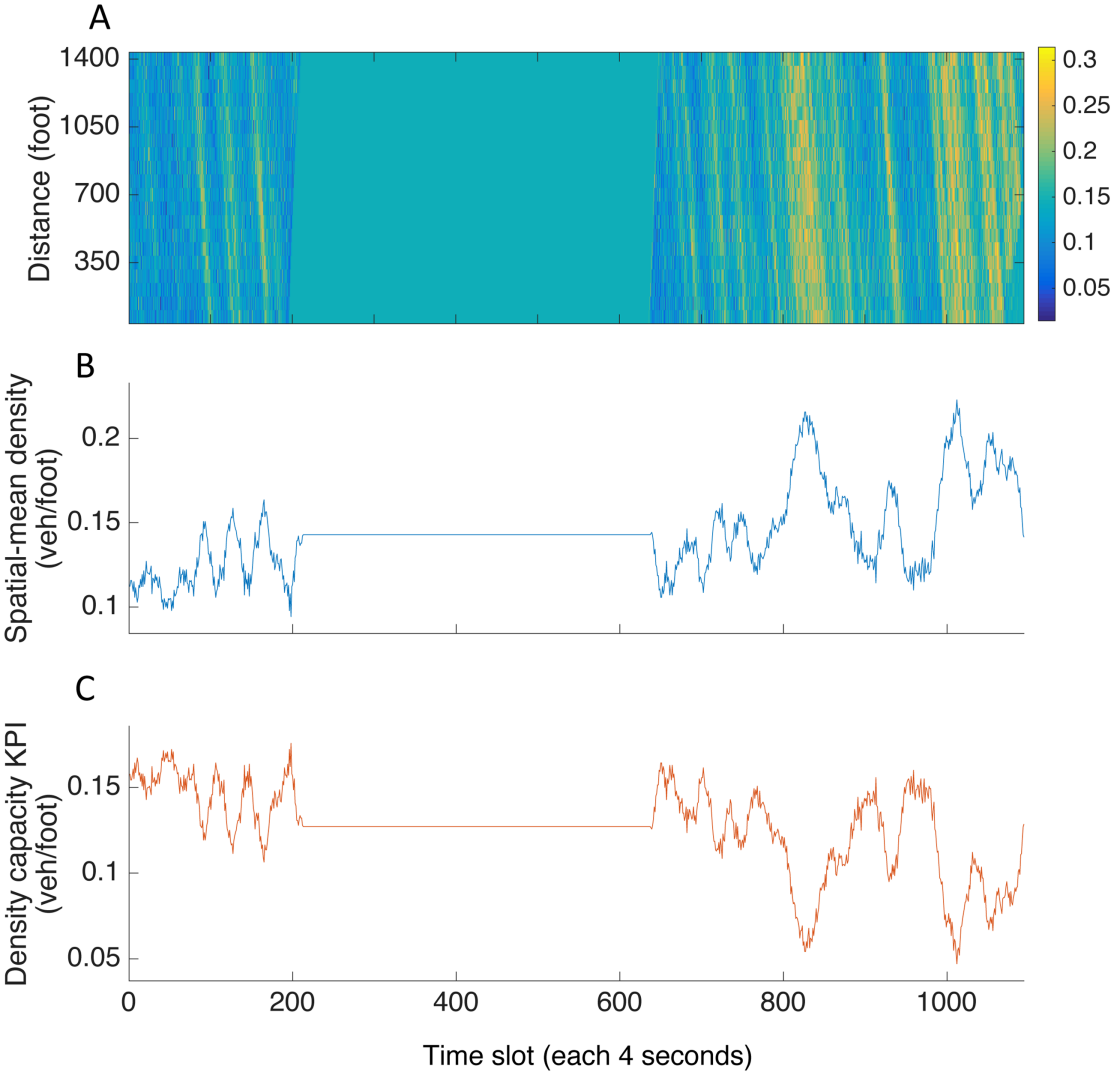
I-80 spatial-temporal pattern and KPI. (A) Full range of spatial-temporal density profile, which contains a 45-minutes time gap in the middle with I-80 16:00-16:15 before the gap and I-80 17:00-17:30 after it. (B) Aggregated spatial-mean density plot. (C) The KPI density capacity, which should be noted that it forms a mirror image with density performance.

Once the KPI is determined, analysis of spatial-temporal patterns can then be conducted accordingly. This technique of analysis is not uncommon in congestion studies, as it is always useful to identify the congestion and offers a direct visualization of traffic conditions within the study area. Fig 4 illustrates the process from construction of spatial-temporal profile to KPI conversion in I-80 case. With clear visual indication, the reconstructed spatial-temporal map enables one to identify jamming patterns quickly and facilitate the further analysis.

### Congestion Index (CI)

We implement and test all metrics on their measuring strength and analyze their comparative performance in this section. The “SGOLAY” algorithm in MATLAB package [43] was applied to smooth and de-noise KPI since vehicle trajectory data usually collected with unavoidable background noise. Prior to identification of draw-down and draw-up cycles, it is better to normalize the KPI to set up a uniform scale so that it falls in the range [0, 1]. Here, normalized KPI was achieved by finding simple statistical normalization of spatial-mean density capacity at each time step, $k_{\left(i\right)}^{\prime }$, to the maximum density capacity, $k_{max}^{\prime }$. Thus, the normalized KPI of study area at time *i* can be realized as $k_{\left(i\right)}^{\prime }/k_{max}^{\prime }$.

The identification of draw-down and draw-up cycles was then conducted according to studies [44, 45] of *ϵ*−filtering algorithm, which detects the significance of upturns and downturns by a constant threshold of *α*% on its magnitude. The reasons for performing such filtering identification process before metric implementation are as follows: (1) The proposed metric is constructed based on draw-down and draw-up cycles, therefore, one should ensure all the cycles identified are representative for each recurrent congestion pattern in spatial-temporal profile; (2) Without *ϵ*− filtering process, it would yield pure draw-downs and draw-ups (every single fluctuation), which is obviously unnecessary for those insignificant oscillations to participate in congestion measurement. However, it still requires the detection of pure downs and ups before conducting *ϵ*− filter. The *α* in *ϵ*− filtering algorithm was set as 50% since we were only interested in significant congestion (i.e., a draw-down/draw-up will only be recognized if its magnitude is more than half of its preceding draw-up/draw-down. A simplified pseudocode is given in S1 Code). Taking I-80 as an illustrative example, the identification process returned 18 recognizable draw-down and draw-up cycles, which indicates 18 congestion patterns were detected.

The initial values of Elasticity Threshold and Robustness Range were determined by critical density *k*_*critical*_, because it is the threshold where phase transition occurs. But for the normalized KPI, those two parameters also need to be normalized to keep consistency on the scale. Recall that the *k*_*critical*_ for I-80 case was determined as 0.15 veh/ft, the density capacity at this threshold *k*′ = *k*_*jam*_ − *k*_*critical*_ = 0.27 − 0.15 = 0.12 veh/ft, and this critical capacity value is then normalized as $\text{ET}=k^{\prime }/k_{max}^{\prime }=0.12/0.18=0.67$ ($k_{max}^{\prime }$ in I-80 is 0.18 veh/ft). And RR was assumed as 10% of ET. Hereafter, ET and RR in US-101 and LB can be determined accordingly and all metrics can be implemented.

**Fig 5:**
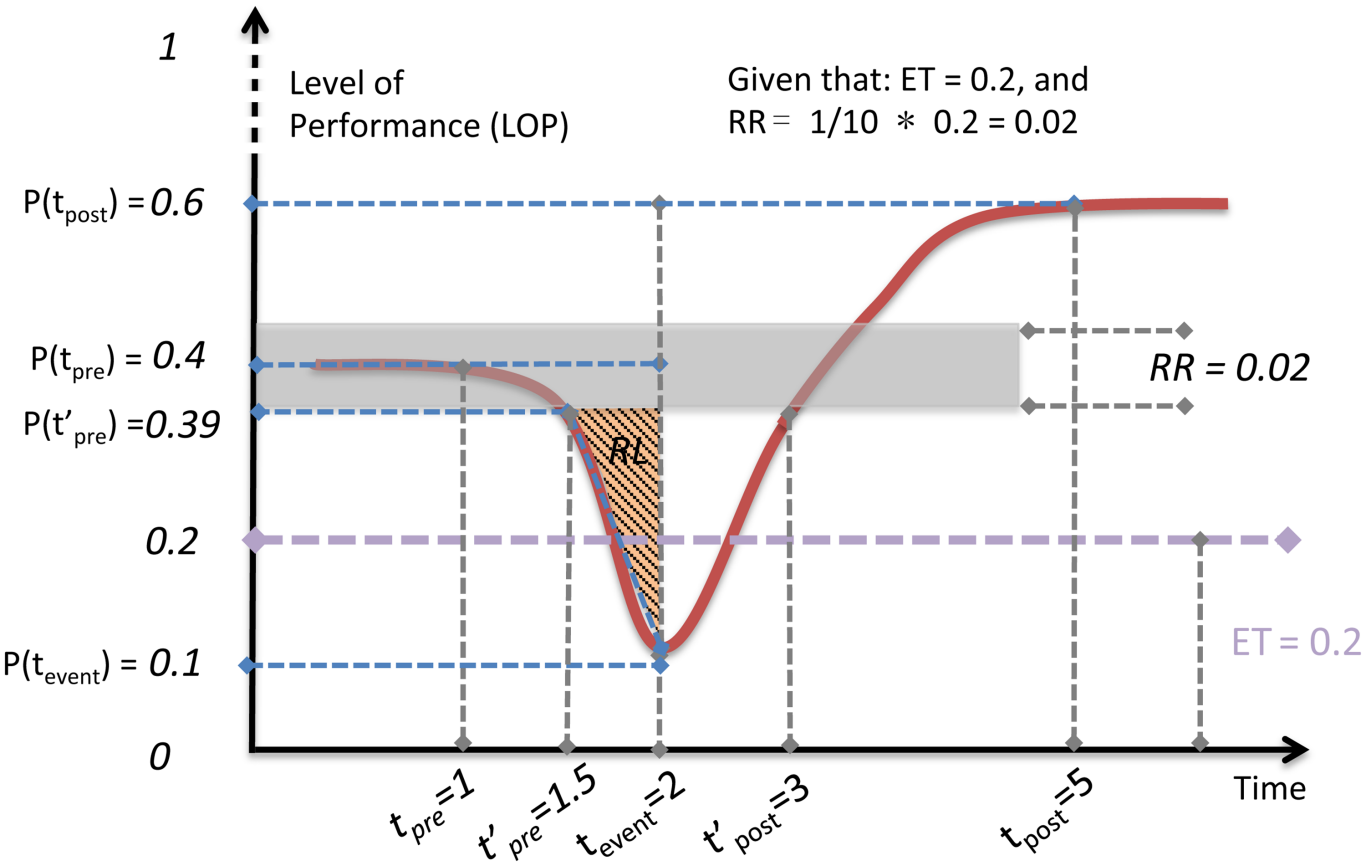
Illustrative example. Demonstration of an adaptive-recovery case for Congestion Index (CI) calculation.

One numeric example of how to calculate CI with proposed metric is given for a step-by-step demonstration in Fig 5. In this illustration, we have ET given as 0.2. All key points for computing each elemental functions in proposed metric are also numerically presented. Therefore, we have:

1. **ET** is given as 0.2. Therefore, **RR** = 1/10 × 0.2 = 0.02.
2. **Congestion Magnitude** $C_{m}=P_{\left(t_{pre}^{\prime }\right)}-P_{\left(t_{event}\right)}=0.39-0.1=0.29$
3. **Congestion Time** $C_{t}=\left(t_{event}-t_{pre}^{\prime }\right)/\left(t_{post}^{\prime }-t_{pre}^{\prime }\right)=\left(2-1.5\right)/\left(3-1.5\right)=0.33$
4. **Recovery Scenario** Because *P*_(*t*_*post*_)_ > *P*_(*t*_*pre*_)_. Then the **Recovery Scenario** *R*_*s*_ = 1 with a positive sign “+”.
5. **Resistance Coefficient** Because *ET* > *P*_(*t*_*event*_)_, *R*_*e*_ = 0.2 − 0.1 = 0.1
6. Overall congestion index for this example cycle is calculated as $\text{CI}=\left(\frac{0.29\times 0.33}{2}+0.1\right)\times \left(+1\right)=+0.148$

**Fig 6:**
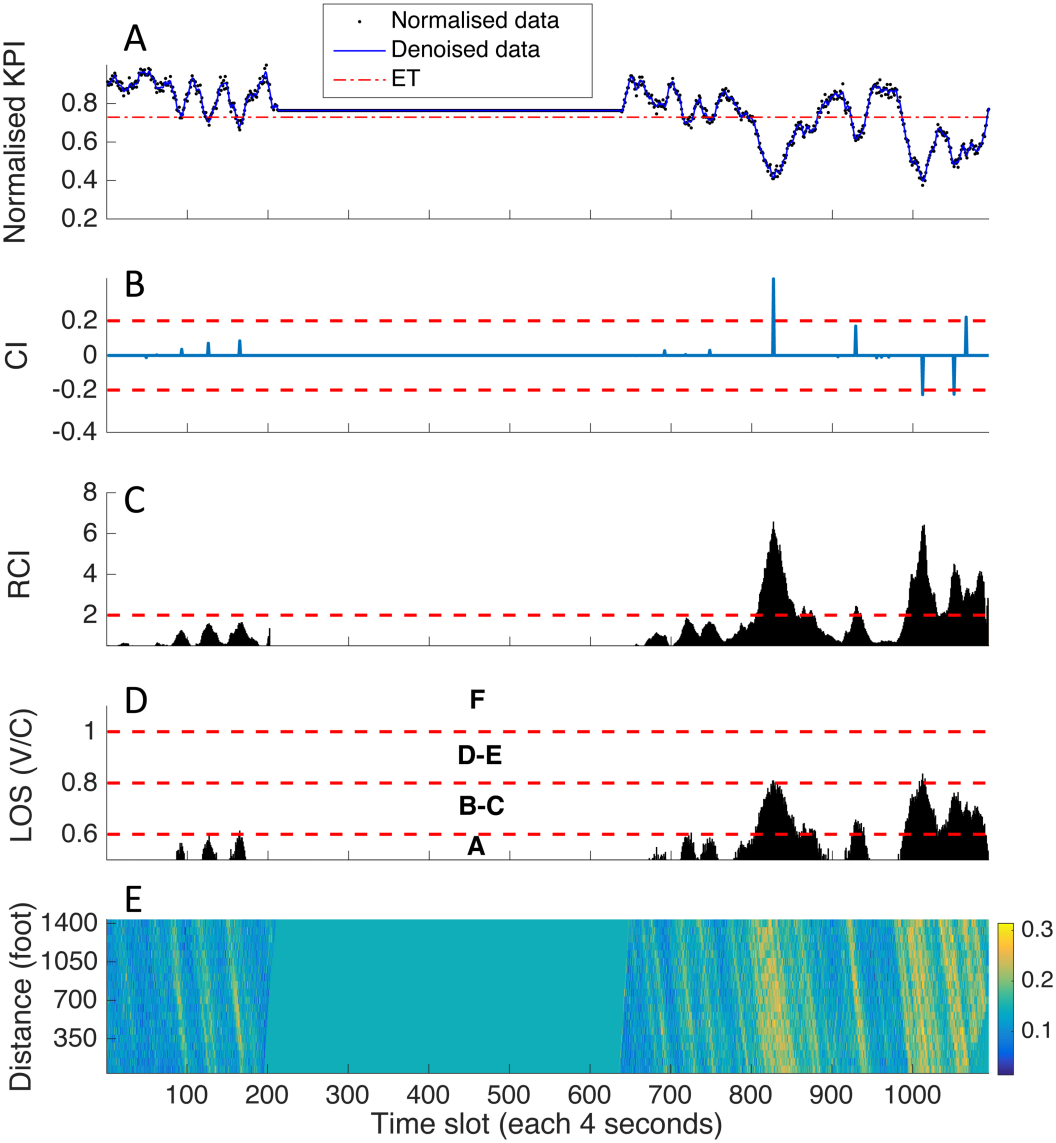
Congestion indexes of I-80. Congestion quantification results of all three metrics compare with the ground-truth pattern. (A) Normalized KPI with *ET* = 0.67. (B) CI. (C) RCI. (D) LoS. (E) Ground-truth pattern. The positive and negative signs denote different *R*_*s*_ in down-and-up cycles, which provide dynamic information about congestion recovery.

Following the normalization illustrated in Fig 6A and recalling Eqs 6 and 9, resultant CI, RCI and LoS for I-80 are shown in Fig 6B–6D with their statistics in Table 3. By comparing with the ground-truth spatial-temporal patterns (Fig 6E), it can be seen that all the significant congestion patterns were captured by CI metric. In order to represent a complete down-and-up cycle and also to capture the local maxima in RCI and LoS results, all the congestion indexes were plotted at *t*_*event*_ in each cycle. In first 200 time steps, there is no severe congestion occurred, as three notable patterns are all indicated as CI less than 0.2, RCI less than 2 and LoS in level A. Nevertheless, at around 800th time steps, several significant congestions occurred as indexes quickly turn to negative readings with increasing intensity over 0.2. It indicates that the traffic condition in latter observation of I-80 (17:00 p.m. to 17:30 p.m.) was far more congested than former 15 minutes (16:00 p.m. to 16:15 p.m.). Moreover, successive and large negative CI denote insufficient discharging processes in these congestion cycles, which further enhance our interpretation of their relative severity.

**Table 3:** The quantification results for 18 draw-down and draw-up cycles and congestion evaluation of all three metrics. The values for RCI and LoS are obtained by finding local maximum at *t*_*event*_.

No.	*R*_*s*_	Time slot at *t*_*event*_	CI	RCI	V/C (LoS)
1	-	49	-0.011	0.321	0.391
2	+	62	0.003	0.618	0.463
3	+	93	0.032	1.248	0.565
4	+	126	0.063	1.534	0.573
5	+	165	0.076	1.600	0.613
6	-	657	-0.001	0.580	0.477
7	+	692	0.025	1.007	0.512
8	+	718	0.005	1.747	0.589
9	+	748	0.027	1.660	0.587
10	+	827	0.398	6.569	0.809
11	-	907	-0.007	0.758	0.477
12	+	929	0.153	2.431	0.656
13	-	955	-0.013	0.629	0.466
14	-	961	-0.009	0.744	0.477
15	-	970	-0.008	0.745	0.485
16	-	1012	-0.204	6.364	0.836
17	-	1051	-0.202	4.501	0.761
18	+	1066	0.199	3.207	0.715

Comparing CI with RCI and LoS at the local maximum at *t*_*event*_ in each cycle, we found the intensity of CI, RCI and LoS have similar indications. What’s different by comparing with latter two metrics is that CI not only provides relative intensity differences among congestion patterns, but also reasonably amplify the scales to differentiate major and minor congestion. For instance, there are three successive jam patterns occurred around 700th time slot, and CI detected them as minor patterns with small values while RCI and LoS assigned relatively high values to them (yet still quantified as un-congested flow by RCI and LoS). A rule-of-thumb judging criterion of CI can be made—that is—patterns will be considered as major congestion when the absolute value of their CI is greater than 0.2.

Most importantly, unlike traditional congestion measure methods, CI metric can also indicate the situation of post-event recovery as well. For instance, those short but negative indications are of particular interest. They indicate small-scale congestion with an insufficient discharging outcome. In other words, the I-80 freeway did not fully recover or completely dissolve the previous congestion queue before next one occurred at that point. Such implication could be hardly identified on spatial-temporal patterns by visual judgment and other conventional metrics, like RCI and LoS. Also, those small but positive indications illustrate immediate congestion formations with quick discharge. Together, they might be prefigured as signs for coming massive jams.

**Fig 7:**
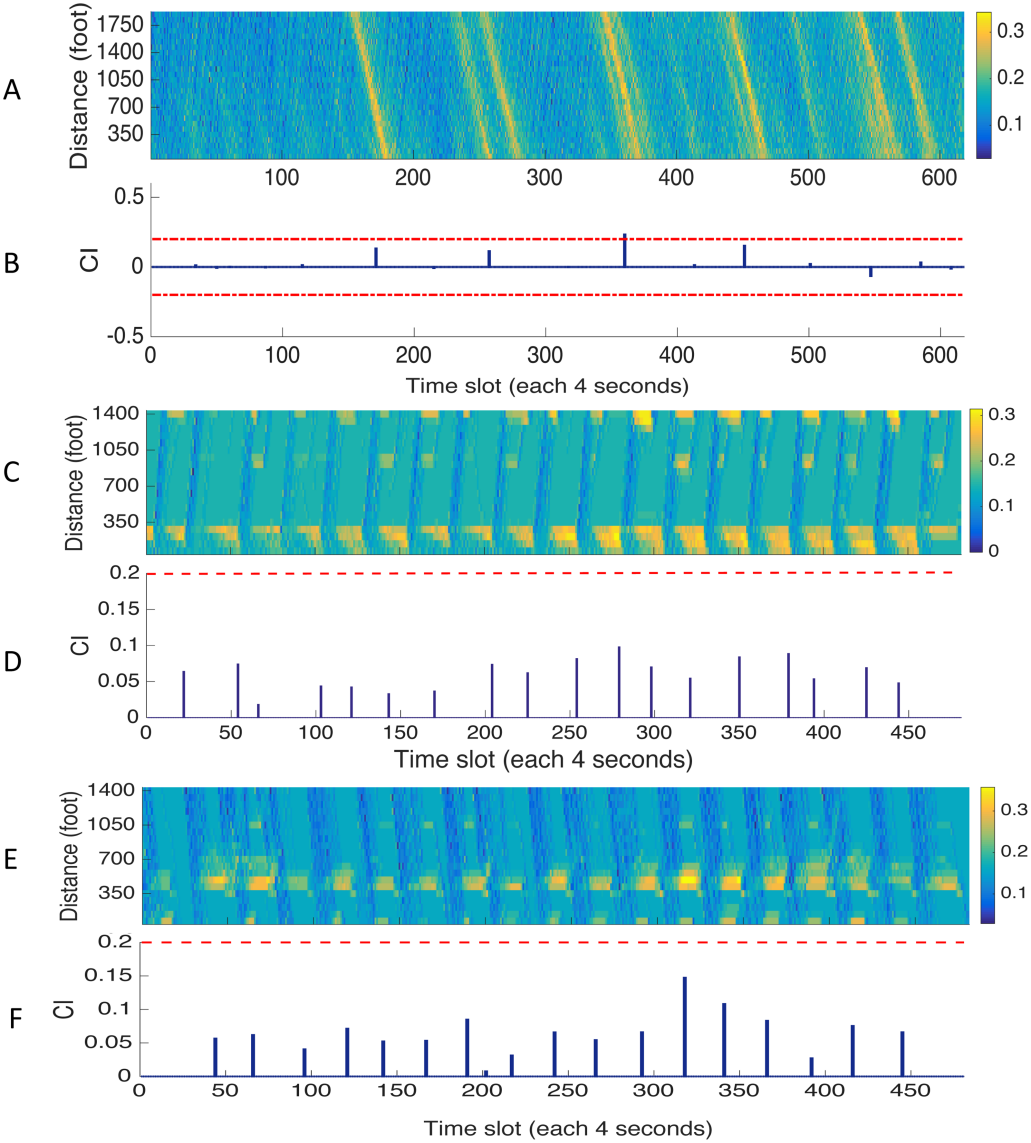
Congestion indexes for US-101 and LB. Results of CI compared with the ground-truth pattern. (A), (C), and (E) are the ground-truth spatial-temporal profiles for US-101, northbound and southbound of LB. (B), (D), and (F) are congestion indexes calculated by proposed metric correspondingly.

Fig 7 demonstrates the CI results for US-101, northbound and southbound of LB cases. Tables 4 and 5 contain the numerical measuring results of all metrics. As illustrated in Fig 7A and 7B, the morning peak-hour traffic was somehow less congested than expected. It may be due to the fact that southbound of US-101 in morning is not in high traffic demand (away from the attractor such as city center). Even so, CI metric still performs well in this case. In Table 4, the absolute intensity of its quantified congestion, again, have similar variations as the outcomes obtained by other two. However, the only difference is that several congestion patterns in CI were not as significant as quantified in RCI and LoS. This could be the result of the observations that US-101 was less saturated and discharging processes of its congestion patterns were rather quick.

**Table 4:** The quantification results for US-101 using all three metrics. The values for RCI and LoS are obtained by finding local maximum at *t*_*event*_.

No.	Time slot at *t*_*event*_	CI	RCI	LoS
1	34	0.008	0.572	0.575 (A)
2	50	-0.007	0.505	0.512 (A)
3	60	0.001	0.594	0.538 (A)
4	87	-0.004	0.420	0.508 (A)
5	115	0.010	0.729	0.544 (A)
6	171	0.129	2.199	0.742 (B)
7	215	-0.008	0.420	0.494 (A)
8	257	0.110	2.078	0.736 (B)
9	317	-0.002	0.606	0.542 (A)
10	360	0.229	3.185	0.793 (B)
11	413	0.010	1.319	0.621 (B)
12	451	0.149	2.586	0.758 (B)
13	501	0.019	1.386	0.647 (B)
14	547	-0.065	2.986	0.789 (B)
15	585	0.029	1.757	0.714 (B)
16	608	-0.012	0.961	0.585 (A)

**Table 5:** The quantification results for Lankershim Boulevard (LB). The values for RCI and LoS are obtained by finding local maximum at *t*_*event*_. 18 recurrent and regularized patterns can be observed in both directions.

Northbound					Southbound			
No.	*t*_*event*_	CI	RCI	LoS	*t*_*event*_	CI	RCI	LoS
1	44	0.061	26.373	0.533 (A)	44	0.056	22.713	0.595 (A)
2	66	0.071	80.114	0.526 (A)	66	0.062	41.509	0.624 (B)
3	96	0.017	11.861	0.531 (A)	96	0.040	3356.005	0.552 (A)
4	121	0.041	25.501	0.524 (A)	121	0.071	1990.326	0.579 (A)
5	142	0.040	29.713	0.543 (A)	142	0.052	82.635	0.571 (A)
6	167	0.032	39.027	0.548 (A)	167	0.053	199.322	0.574 (A)
7	191	0.035	14.980	0.533 (A)	191	0.085	20.544	0.581 (A)
8	202	0.070	24.187	0.521 (A)	202	0.008	1.146	0.536 (A)
9	217	0.060	31.177	0.552 (A)	217	0.031	8.181	0.567 (A)
10	242	0.078	194.181	0.548 (A)	242	0.066	20.568	0.598 (A)
11	266	0.095	1618.633	0.567 (A)	266	0.054	13.711	0.583 (A)
12	293	0.068	61.873	0.598 (A)	293	0.066	25.947	0.598 (A)
13	318	0.053	74.996	0.610 (B)	318	0.147	1467.761	0.643 (B)
14	341	0.082	23.220	0.576 (A)	341	0.108	36.551	0.629 (B)
15	366	0.086	1226.453	0.552 (A)	366	0.083	29.909	0.614 (B)
16	392	0.052	66.122	0.569 (A)	392	0.027	905.763	0.631 (B)
17	416	0.067	15.108	0.574 (A)	416	0.075	72.630	0.617 (B)
18	445	0.047	26.754	0.586 (A)	445	0.066	998.680	0.574 (A)

The results from LB cases show interesting features (Fig 7C–7F). Because it is a section of an urban arterial with signal-controlled junctions and mixed groups of road users, regularized jam patterns can be clearly spotted. One may also notice the directions of propagation waves on two bounds are distinct. Even so, CI metric showed adequate measuring strength to characterize recurrent and controlled congestion patterns. Overall, the spatial-mean traffic condition on LB was unsaturated without residual queues. In contrast, RCI performs badly in this case as the values obtained at local maxima are dramatically high as shown in Table 5. This could be a result of regularized traffic on this type of road. Signal-controlled junctions signify that the spatial-mean speed along study area could be extremely small at some time step if most of the vehicles were stopped by junction signals, and this leads to very high values of *T*_*ac*_ in Eq 7. Since *T*_*ff*_ is constant, the RCI could have a very large value when *T*_*ac*_ is large, and therefore, makes the indexes unrepresentative for actual overall traffic condition in the study area. This exactly proves its shortcoming mentioned in the previous section.

### Sensitivity analysis

Sensitivity results of three critical parameters in the metric and different cell size are evaluated in this subsection. Since the metric heavily rely on the determination of parameters, the sensitivity of *ϵ*, RR, ET and cell size need to be studied. There are two facets in this analysis as we want to know how variation of these parameters affect (1) the number of congestion cycles detected, and (2) the measured absolute intensity of congestion.

**Fig 8:**
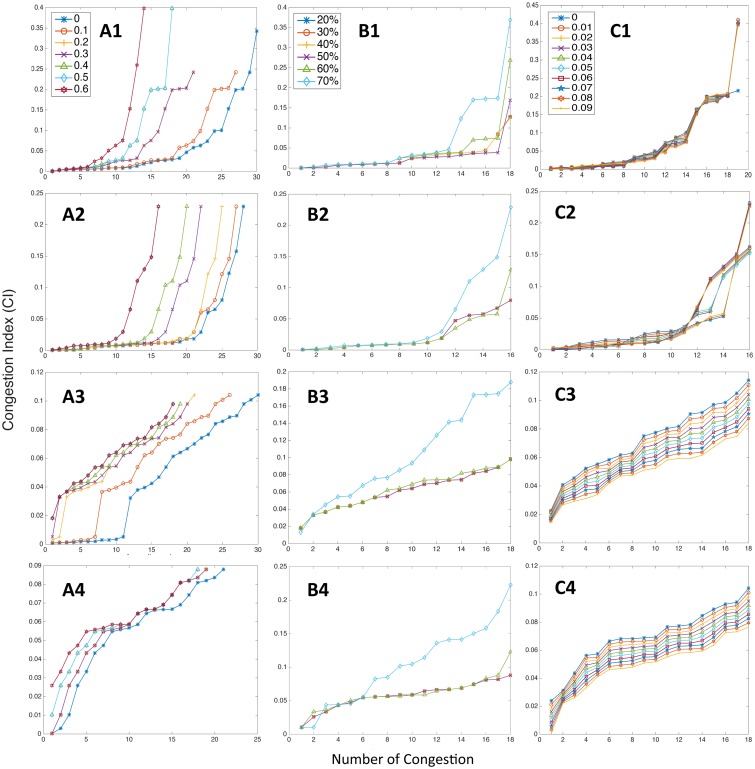
Sensitivity analysis on *ϵ*, ET and RR values. (A) Sensitivity test on *ϵ*. (B) Test on ET, and (C) Test on RR. (1) Case I-80. (2) Case US-101. (3) Northbound of LB, and (4) Southbound of LB.

We tested the *ϵ* from 0 to 0.6, i.e., from pure draw-down and draw-up to 60% of filtering threshold. By sorting the absolute values of congestion indexes in ascending order, Fig 8A1–8A4 illustrate that the number of congestion index is significantly affected by *ϵ* (as the value of it increases, the number of identified cycles decreases). However, the scales of the indexes show low sensitivity to variation once the *ϵ* is established, especially the major congestion, the change in *ϵ* does not significantly alter the detection of those major congestion and their scales roughly remain stable.

Interestingly when it comes to the test on Elasticity Threshold (ET), results show high sensitivity to small variation of ET (from 0.2 to 0.7). In Fig 8B1–8B4 the difference between major and minor congestion indexes, in the beginning, is hardly detected, it makes sense since small ET indicates that no phase transition occurred. And with increasing ET, the difference starts to be revealed. It verifies that the existence of phase transition is vital in quantification process, especially for identifying and differentiating major patterns. On the other hand, ET has no effect on the number of cycles detected.

The overall scale of indexes shows a low sensitivity to the variation of Robustness Range (RR) from 0 to 0.09 (1/10 of ET) in Fig 8C1–8C4. As can be seen, the measuring strength of our proposed metric is not dramatically sensitive to RR. But we can also observe different features from Fig 8C3 and 8C4, the intensity of indexes gradually decreases as RR increases. This is due to the regularized feature in controlled traffic as all congestion cycles have similar depth and shape so that increasing amount of RR causes a similar amount of deduction on *C*_*m*_. Meanwhile, RR cannot influence the detected number either.

**Fig 9:**
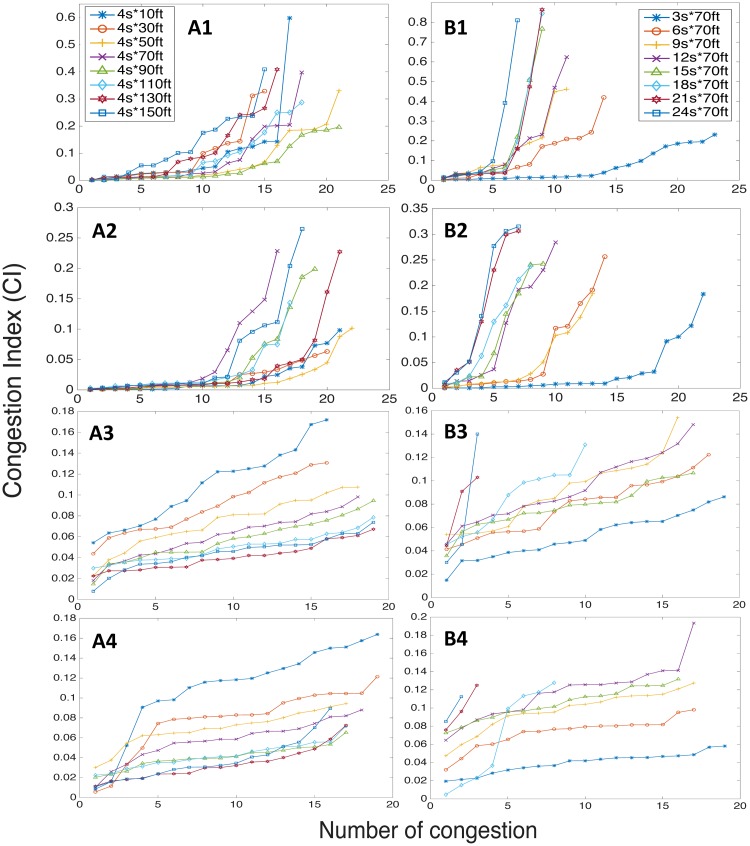
Sensitivity analysis on cell size. (A) Sensitivity test on spatial dimension with constant temporal length. (B) Sensitivity test on temporal dimension with constant spatial length. (1) I-80. (2) US-101. (3) Northbound of LB, and (4) Southbound of LB.

Fig 9 demonstrates the sensitivity results on cell size. From Fig 9A1–9A4 analysis were performed based on changing spatial length but keeping a constant temporal length, and Fig 9B1–9B4 were, in contrast, subjected to changing temporal length with a constant spatial length. There is a common pattern throughout all four cases, which indicates that a small dimensional change of cell size could drastically affect the measuring outcomes on both facets. One can see that with changes on spatial length as from 4 seconds × 10 feet (approx. 3.05m) to 4 seconds × 150 feet (approx. 45.72m), the absolute intensity of detected congestion were constantly shifting. From both freeway and arterial cases, we can see that spatial length of cell tends to have relatively more sensitive leaps on the intensity of CI rather than the number. However, in Fig 9B1–9B4 all cases show relatively high sensitivity on both number and intensity of indexes to the variation in temporal length. For instance, only a few cycles can be identified when the temporal length is 24 seconds in LB cases. This could possibly imply that, with too small cell size, too many frivolous fluctuation details were captured and they influence the overall measuring outcomes with an unrepresentative number of vehicles in each cell. On the other hand, some congestion wave would be missed out if the cell size is too large, causing dropping number of identified congestion cycles. Also, the metric implementation outcome seems to be more sensitive to the temporal length of cells since the traffic patterns were studied in spatial-mean along the temporal dimension.

## Discussion and conclusion

There are some potential limitations about this metric and the vehicle trajectory data used [46, 47]. Traffic operators should be particularly aware of these limitations in practice.

- Data availability and type, such as dirty and mutilated data, would significantly influence implementation of the metric. As we found during the tests, inactive cells in spatial-temporal profile could alter the outline of the spatial-mean density capacity. Such attribute requires a good data treatment which could limit potential applications of the metric. For example, if the trajectory data is collected from GPS or other types of onboard mobile sensors, a bad penetration or sampling rate could destabilize the metric performance.
- The initial implementation of CI metric involves multiple steps and can be potentially complex for non-technical users. However, similar limitations are often solved by a proper built-in function in tools. We found that the processing time of the whole experiment is heavily depending on the input data but the processing time of metric per se in a total run is rather quick.
- Both temporal and spatial coverage of study areas are insufficient, especially considering the fact that a peak hour usually lasts for longer period of time, and congestion propagation could also last for longer distance.
- The type of roads and traffic conditions are limited. The datasets merely cover US freeways and urban arterials, leading to impossible investigations on other road types or in other countries. Furthermore, the traffic in US-101 and LB lack saturated conditions, which lead to a lot of uncertainty on metric compatibility in extreme traffics.

In conclusion, our study addressed the issue of quantifying recurrent congestion based on spatial-temporal patterns on both urban freeways and streets. We constructed a metric inspired by the principle of well-applied “R4 resilience-triangle” approach, with the goal of quantitatively assessing and comparing congestion occurred repeatedly in various temporal steps. The representativeness of the metric and associated generic dimensions presented a strong capability for quantification and assessment. Our main conclusions are summarized below.

The resilience-based approach provides a unique and different angle for tackling the congestion quantification issue, and its new-build characteristic dimensions are effective for capturing and differentiating major congestion. The signs of congestion indexes (positive or negative based on recovery performance) illustrate not only the overall congestion intensity but also indicate the discharging process after its formation. Our study amplifies the congestion quantification toolbox and establishes a combination of system resilience analysis.

The proposed metric shows relative merits in measuring and characterizing strength as compared with other two traditional metrics, RCI and LoS. Because the construction of the metric is based on generic traffic dimensions, it has been found to be applicable to both freeway and arterial cases. The metric performs adequately in signal-controlled traffic and outperforms RCI as shown in Lankershim Boulevard case.

Sensitivity tests verify that the phase transition mechanism plays an indispensable role in congestion analysis as the metric showed sensitive behavior to the Elasticity Threshold (ET). The testing results on *ϵ* and RR show relatively low sensitivity on detecting major congestion but the number of identified congestion patterns can be influenced by the existence of *ϵ*. The tests on various cell size demonstrate a sensitive behavior of metric to its discrete platform construction. Particularly we found that both number and intensity of detected congestion patterns are highly sensitive to the spatial dimension.

This study provides insights into the quantification of recurrent traffic congestion inspired by emerging resilience concept. The metric we constructed showed strength in quantitative analysis of congestion in a systemic perspective and potentially offers an alternative for congestion study across different scenarios. Future research will further investigate the application of the metric to various traffic conditions in other countries and expand understandings of its application in road networks.

## Supporting information

S1 Code Pseudocode for *ϵ*−filtering algorithm. (PDF)

## References

[1] Lomax TJ. Quantifying congestion. No. 398. Transportation Research Board; 1997.

[2] Hall RW. Non-recurrent congestion: how big is the problem? Are traveler information systems the solution? Transp Res Part C Emerg Technol. 1993;1(1):89–103. 10.1016/0968-090X(93)90022-8.

[3] Rao AM, Rao KR. Measuring urban traffic congestion—A review. International Journal for Traffic & Transport Engineering. 2012;2(4):286–305. 10.7708/ijtte.2012.2(4).01.

[4] Lindley JA. Urban freeway congestion: quantification of the problem and effectiveness of potential solutions. ITE journal. 1987;57(1):27–32.

[5] Lindley JA. Quantification of urban freeway congestion and analysis of remedial measures. Final report. Available from: https://trid.trb.org/view.aspx?id=285553; 1986.

[6] d’Abadie RR, Ehrlich TF. Contrasting time-based and distance-based measures for quantifying traffic congestion levels analysis of New Jersey counties. Transp Res Rec. 2002;(1817):143–148. 10.3141/1817-18.

[7] Milojevic M, Rakocevic V. Distributed road traffic congestion quantification using cooperative VANETs. In: Ad Hoc Networking Workshop (MED-HOC-NET), 2014 13th Annual Mediterranean. IEEE; 2014. p. 203–210. 10.1109/MedHocNet.2014.6849125.

[8] Armah FA, Yawson DO, Pappoe AA. A systems dynamics approach to explore traffic congestion and air pollution link in the city of Accra, Ghana. Sustainability. 2010;2(1):252–265. 10.3390/su2010252.

[9] Kerner BS. Empirical macroscopic features of spatial-temporal traffic patterns at highway bottlenecks. Phys Rev E. 2002;65(4):046138 10.1103/PhysRevE.65.046138. 12005957

[10] Kerner BS. Control of spatiotemporal congested traffic patterns at highway bottlenecks. IEEE trans Intell Transp Syst. 2007;8(2):308–320. 10.1109/TITS.2007.894192.

[11] Kerner BS, Rehborn H, Aleksic M, Haug A. Recognition and tracking of spatial–temporal congested traffic patterns on freeways. Transp Res Part C Emerg Technol. 2004;12(5):369–400. 10.1016/j.trc.2004.07.015.

[12] Yuan J, Mills K. A cross-correlation-based method for spatial–temporal traffic analysis. Performance evaluation. 2005;61(2):163–180. 10.1016/j.peva.2004.11.003.

[13] Knospe W, Santen L, Schadschneider A, Schreckenberg M. Towards a realistic microscopic description of highway traffic. J Phys A Math Gen. 2000;33(48):L477 10.1088/0305-4470/33/48/103.

[14] Jianming H, Qiang M, Qi W, Jiajie Z, Yi Z. Traffic congestion identification based on image processing. IET Intelligent Transport Systems. 2012;6(2):153–160. 10.1049/iet-its.2011.0124.

[15] Davies P. Assessment of advanced technologies for relieving urban traffic congestion. 340. Transportation Research Board, National Research Council; 1991.

[16] Young T. A course of lectures on natural philosophy and the mechanical arts. vol. 2. Johnson; 1807.

[17] Hollnagel E, Woods DD, Leveson N. Resilience engineering: Concepts and precepts. Ashgate Publishing, Ltd; 2007.

[18] Holland JH. Complex adaptive systems. Daedalus. 1992; p. 17–30. http://www.jstor.org/stable/20025416.

[19] Holling CS. Resilience and stability of ecological systems. Annu Rev Ecol Syst. 1973; p. 1–23. 10.1146/annurev.es.04.110173.000245.

[20] Ong AD, Bergeman C, Bisconti TL, Wallace KA. Psychological resilience, positive emotions, and successful adaptation to stress in later life. J Pers Soc Psychol. 2006;91(4):730 10.1037/0022-3514.91.4.730. 17014296

[21] Tugade MM, Fredrickson BL, Feldman Barrett L. Psychological resilience and positive emotional granularity: Examining the benefits of positive emotions on coping and health. J Pers. 2004;72(6):1161–1190. 10.1111/j.1467-6494.2004.00294.x. 15509280 PMC1201429

[22] Miller JH, Page SE. Complex adaptive systems: An introduction to computational models of social life. Princeton university press; 2009.

[23] Fisher L. Disaster responses: More than 70 ways to show resilience. Nature. 2015;518(7537):35–35. 10.1038/518035a. 25652986

[24] Hosseini S, Barker K, Ramirez-Marquez JE. A review of definitions and measures of system resilience. Reliability Engineering & System Safety. 2016;145:47–61. 10.1016/j.ress.2015.08.006.

[25] Bruneau M, Chang SE, Eguchi RT, Lee GC, O’Rourke TD, Reinhorn AM, et al A framework to quantitatively assess and enhance the seismic resilience of communities. Earthq Spectra. 2003;19(4):733–752. 10.1193/1.1623497.

[26] Tierney K, Bruneau M. Conceptualizing and measuring resilience: A key to disaster loss reduction. TR news. 2007;(250):14–15. 10.17226/23168.

[27] Cimellaro GP, Reinhorn AM, Bruneau M. Framework for analytical quantification of disaster resilience. Eng Struct. 2010;32(11):3639–3649. 10.1016/j.engstruct.2010.08.008.

[28] Administration FH. Next Generation Simulation Factsheet. U.S. Department of Transportation; 2005. Available from: http://ops.fhwa.dot.gov/trafficanalysistools/ngsim.htm.

[29] Administration FH. I-80 Metadata Documentation. U.S. Department of Transportation; 2005. Available from: https://www.its-rde.net/index.php?option=com_joodb&view=article&joobase=8&id=10893&Itemid=218&search=10893&searchfield=dataset_id&deid=10023&detitle=Next%20Generation%20Simulation%20(NGSIM).

[30] Administration FH. US 101 Metadata Documentation. U.S. Department of Transportation; 2005. Available from: https://www.its-rde.net/index.php?option=com_joodb&view=article&joobase=8&id=10892&Itemid=218&search=10892&searchfield=dataset_id&deid=10023&detitle=Next%20Generation%20Simulation%20(NGSIM).

[31] Administration FH. Lankershim Blvd Metadata Documentation. U.S. Department of Transportation; 2005. Available from: https://www.its-rde.net/index.php?option=com_joodb&view=article&joobase=8&id=10894&Itemid=218&search=10894&searchfield=dataset_id&deid=10023&detitle=Next%20Generation%20Simulation%20(NGSIM).

[32] Daganzo CF, Cassidy MJ, Bertini RL. Possible explanations of phase transitions in highway traffic. Transp Res Part A Policy Pract. 1999;33(5):365–379. 10.1016/S0965-8564(98)00034-2.

[33] Kerner BS. The physics of traffic: empirical freeway pattern features, engineering applications, and theory. Springer; 2012.

[34] Kerner BS. Introduction to modern traffic flow theory and control: the long road to three-phase traffic theory. Springer Science & Business Media; 2009.

[35] Downs A. Still stuck in traffic: coping with peak-hour traffic congestion. Brookings Institution Press; 2005.

[36] Kumar SV, Sivanandan R. Congestion Quantification Measures and their Applicability to Indian Traffic Conditions. In: Proceedings of International Conference on Advances in Architecture and Civil Engineering (AARCV 2012). vol. 21; 2012. p. 526. Available from: http://www.conference.bonfring.org/papers/MSR_AARCV2012/TRA120.pdf

[37] Board TR. Highway Capacity Manual. Special report 209, Washington, DC. 1994;1:985.

[38] Aftabuzzaman M. Measuring traffic congestion-a critical review. In: Australasian Transport Research Forum (ATRF), 30TH; 2007. Available from: http://atrf.info/papers/2007/2007_Aftabuzzaman.pdf

[39] Zhang H. Link-journey-speed model for arterial traffic. Transportation Research Record: Journal of the Transportation Research Board. 1999;(1676):109–115. 10.3141/1676-14

[40] Kerner BS. Three-phase traffic theory and highway capacity. Physica A: Statistical Mechanics and its Applications. 2004;333:379–440. 10.1016/j.physa.2003.10.017

[41] Parmenter D. Key performance indicators: developing, implementing, and using winning KPIs. John Wiley & Sons; 2015.

[42] Fitz-Gibbon CT. Performance indicators. vol. 2 Multilingual Matters; 1990.

[43] Mathworks. Filtering and Smoothing Data; 2016. Available from: http://www.mathworks.com/help/curvefit/smoothing-data.html [cited 30/07/2017].

[44] Filimonov V, Sornette D. Power law scaling and “Dragon-Kings” in distributions of intraday financial drawdowns. Chaos Solitons Fractals. 2015;74:27–45. 10.1016/j.chaos.2014.12.002.

[45] Johansen A, Sornette D. Large stock market price drawdowns are outliers. Journal of Risk. 2000;4:69–110. 10.2139/ssrn.244563.

[46] He Z. Research based on high-fidelity NGSIM vehicle trajectory datasets: A review. 2017. 10.13140/RG.2.2.11429.60643

[47] Sun Z, Ban XJ. Vehicle trajectory reconstruction for signalized intersections using mobile traffic sensors. Transportation Research Part C: Emerging Technologies. 2013;36:268–283. 10.1016/j.trc.2013.09.002

