# Supplementary material for: A resilience-oriented approach for quantitatively assessing recurrent spatial-temporal congestion on urban roads
Source: PLoS One. 2018 Jan 2;13(1):e0190616. doi: 10.1371/journal.pone.0190616 (PMC5749843; doi:10.1371/journal.pone.0190616)
Supplement: S1 Code — (PDF) [file pone.0190616.s001.pdf]

In the case of $\varepsilon-$filtering process, a threshold ε must be established for filtering draw-downs and draw-ups. If ε is taken as $\alpha\%$, then accumulations when counting draw-down portions will not stop unless the successive draw-up trend has an upward height that exceeds $\alpha\%$ of the diving depth of previous draw-down. Therefore, when ε is 0, the result of $\varepsilon-$draw-downs filtering is the *pure*−draw-downs, i.e., it captures every detailed fluctuation.

```
begin
    for i= from the first pair to the last do
        if draw-down is greater than draw-up then
            if draw-up is greater than α% of draw-down then
                keep this draw-up;
            else
                ignore this small draw-up and emerge the draw-downs with successive
                draw-down
            end
        else
            (draw-down is smaller than draw-up);
            if draw-down is greater than α% of draw-up then
                keep this draw-down;
            else
                ignore this small draw-down and emerge the draw-up with successive
                draw-up
            end
        end
    end
    return results;
end
```

Algorithm 1: The pseudocode of algorithm to identify $\varepsilon-$draw-downs and $\varepsilon-$draw-ups

Remark. *α is an integer greater than 0*
